# Integrated Assessment of Circulating Cell-Free MicroRNA Signatures in Plasma of Patients with Melanoma Brain Metastasis

**DOI:** 10.3390/cancers12061692

**Published:** 2020-06-25

**Authors:** Matias A. Bustos, Kevin D. Tran, Negin Rahimzadeh, Rebecca Gross, Selena Y. Lin, Yoshiaki Shoji, Tomohiro Murakami, Christine L. Boley, Linh T. Tran, Hunter Cole, Daniel F. Kelly, Steven O’Day, Dave S. B. Hoon

**Affiliations:** 1 Department of Translational Molecular Medicine, John Wayne Cancer Institute at Providence Saint John’s Health Center, Santa Monica, CA 90404, USA; Bustosm@jwci.org (M.A.B.); Negin.Rahimzadeh@providence.org (N.R.); Rebecca.Gentry@providence.org (R.G.); Selena.Lin@providence.org (S.Y.L.); Yoshiaki.Shoji@providence.org (Y.S.); Tomohiro.Murakami@providence.org (T.M.); 2 Department of Genomic Sequencing Center, John Wayne Cancer Institute at Providence Saint John’s Health Center, Santa Monica, CA 90404, USA; Kevin.Tran@providence.org (K.D.T.); linh.tran3@providence.org (L.T.T.); 3 Department of Immuno-Oncology and Clinical Research, John Wayne Cancer Institute, Santa Monica, CA 90404, USA; christine.boley@providence.org (C.L.B.); Hunter.Cole@providence.org (H.C.); O’; 4 Pacific Neuroscience Institute, John Wayne Cancer Institute, Saint John’s Health Center, Santa Monica, CA 90404, USA; kellyd@jwci.org

**Keywords:** cell-free miRNA, melanoma, brain metastasis, plasma, urine, blood, NGS, glioblastoma, immunotherapy

## Abstract

Primary cutaneous melanoma frequently metastasizes to distant organs including the brain. Identification of cell-free microRNAs (cfmiRs) found in the blood can be used as potential body fluid biomarkers for detecting and monitoring patients with melanoma brain metastasis (MBM). In this pilot study, we initially aimed to identify cfmiRs in the blood of MBM patients. Normal donors plasma (healthy, *n* = 48) and pre-operative MBM patients’ plasma samples (*n* = 36) were compared for differences in >2000 microRNAs (miRs) using a next generation sequencing (NGS) probe-based assay. A 74 cfmiR signature was identified in an initial cohort of MBM plasma samples and then verified in a second cohort of MBM plasma samples (*n* = 24). Of these, only 58 cfmiRs were also detected in MBM tissues (*n* = 24). CfmiR signatures were also found in patients who have lung and breast cancer brain metastasis (*n* = 13) and glioblastomas (*n* = 36) compared to MBM plasma samples. The 74 cfmiR signature and the latter cfmiR signatures were then compared. We found a 6 cfmiR signature that was commonly upregulated in MBM plasma samples in all of the comparisons, and a 29 cfmiR signature that distinguishes MBM patients from normal donors’ samples. In addition, we assessed for cfmiRs in plasma (*n* = 20) and urine (*n* = 14) samples collected from metastatic melanoma patients receiving checkpoint inhibitor immunotherapy (CII). Pre- and post-treatment samples showed consistent changes in cfmiRs. Analysis of pre- and post-treatment plasma samples showed 8 differentially expressed (DE) cfmiRs that overlapped with the 35 cfmiR signature found in MBM patients. In paired pre-treatment plasma and urine samples receiving CII 8 cfmiRs overlapped. This study identified specific cfmiRs in MBM plasma samples that may potentially allow for assessment of melanoma patients developing MBM. The cfmiR signatures identified in both blood and urine may have potential utility to assess CII responses after further validation.

## 1. Introduction

Cutaneous melanoma is one of the most aggressive metastatic solid tumors, with an increasing incidence over the last decade [1]. Metastatic melanoma is difficult to manage because of its rapid growth and propensity to spread to distant organs such as the liver, lung, and brain [2]. Melanoma has the third highest incidence of brain metastasis of extracranial origin tumors after lung and breast cancers [3]. Melanoma brain metastasis (MBM) can be lethally aggressive although often remains dormant in early stages and clinically undetectable [4]. New effective checkpoint inhibitor immunotherapies (CII) have significantly improved survival outcomes for metastatic melanoma patients [5,6]; however, early detection of MBMs remain a key factor for the implementation of treatment regimens such as: targeted therapy, CII, radiological, and surgical interventions [5,7]. Also, there is still an issue when evaluating which metastatic patients will benefit from specific therapies [8]. Moreover, there is a lack of blood biomarkers available to monitor metastatic melanoma patients undergoing treatment. Until now, elevated serum lactate dehydrogenase (sLDH) levels are the only validated independent diagnostic blood biomarker for the American Joint Committee on Cancer (AJCC) stage IV melanoma patients, but often sLDH does not translate into meaningful clinical information that can be used for deciding patients’ therapeutic treatments [9,10].

Blood and urine biomarkers such as cell-free nucleic acids (cfNA) are among the most practical, cost-effective, and minimally invasive approaches that allow for easier compliance and participation by patients [11,12]. As biomarkers, cfNA have limitations in solid tumor patients such as: (1) Detection efficacy as related to the tumor burden, (2) amount of specific tumor cfNA that are detectable in the volume sampled, (3) profiling large numbers of cfNA at one time, and (4) degradation of cfNA [13,14]. Detection of metastatic melanoma has improved in recent years, particularly using circulating cell-free DNA (cfDNA) analysis targeting specific mutations [15,16,17,18,19]. Nonetheless, detection of metastatic cutaneous melanomas has been difficult due to a limited number of frequent mutations that are observed at specific gene exon sites, other than *BRAF* (<60%) and *NRAS* (<25%) genes, thus reducing the sensitivity of melanoma detection in blood. Moreover, cfDNA is unstable in body fluids and has a short half-life [20,21]. 

On the contrary, microRNAs (miRs) are stable short sequence (18–22 nucleotides) regulators that exert post-transcriptional control over gene expression [22,23]. Hanniford et al demonstrated that a 4-miR signature is sufficient to discriminate primary melanoma tumors that will metastasize to the brain from those that will not [24]. Also, specific cfmiRs are found in both serum and plasma of cutaneous melanoma patients as our group and others have demonstrated [23,25,26,27,28,29,30,31,32,33,34,35,36]. Several cfmiRs have been proposed as single biomarkers, or in combination to be used for diagnostic and prognostic assessment of cutaneous melanoma patients [23,37]. Nonetheless, there are concerns that these cfmiRs are non-cancer related and are associated with normal physiological functions and benign diseases [23,38]. 

The major issues in the application of cfmiR assays are specificity, reproducibility, and accuracy. The main technical concerns of miR assays are: (1) Inconsistencies in study design where the cfmiR signatures have not been validated in matched tissue samples; (2) fluid sampling volume and efficient extraction of these small cfmiRs; (3) reproducibility and robustness; and (4) detection of low level cfmiRs [23,39,40]. To avoid these issues of cfmiR detection, we used a targeted probe, next generation sequencing (NGS)-based platform (HTG EdgeSeq miRNA Whole Transcriptome Assay (WTA), HTG Edge System software Host version 5.3.772.5028 and HTG REVEAL software version 2.0.1, HTG Molecular Diagnostic, Inc. Tucson, AZ, USA). HTG miRNA WTA profiles the expression of 2083 miRs, requires minimal sample input (<50 µL), has low processing cost, yields high reproducibility, and offers robustness and high accuracy [24]. This platform is based on an extraction-free direct assay that significantly increases the efficiency of capturing low level miRs; therefore reducing data variability introduced by nucleic acid isolation techniques, which is a major problem in cfNA blood assay reproducibility [24,41,42]. More importantly, miRs can be assessed from limited specimen sources.

This study revealed a cfmiR signature found in the plasma of MBM patients, verified in a second MBM cohort, and corroborated in MBM tissues. We demonstrated that the cfmiR signature detected in the MBM patients overlap with the cfmiR found in pre-treatment plasma samples from metastatic melanoma patients receiving CII. CfmiR detection in urine offers a non-invasive body fluid assay that is more logistically practical for patient compliance. Therefore, we have developed a cfmiR assay for evaluating urine to complement the plasma cfmiR assessment of CII-treated melanoma patients. Specific cfmiRs that were upregulated in the plasma were consistently detected in the paired urine samples of CII-treated melanoma patients.

## 2. Results

### 2.1. CfmiRs That Differentiate Plasma from Serum in Normal Healthy Donors

**Figure 1:**
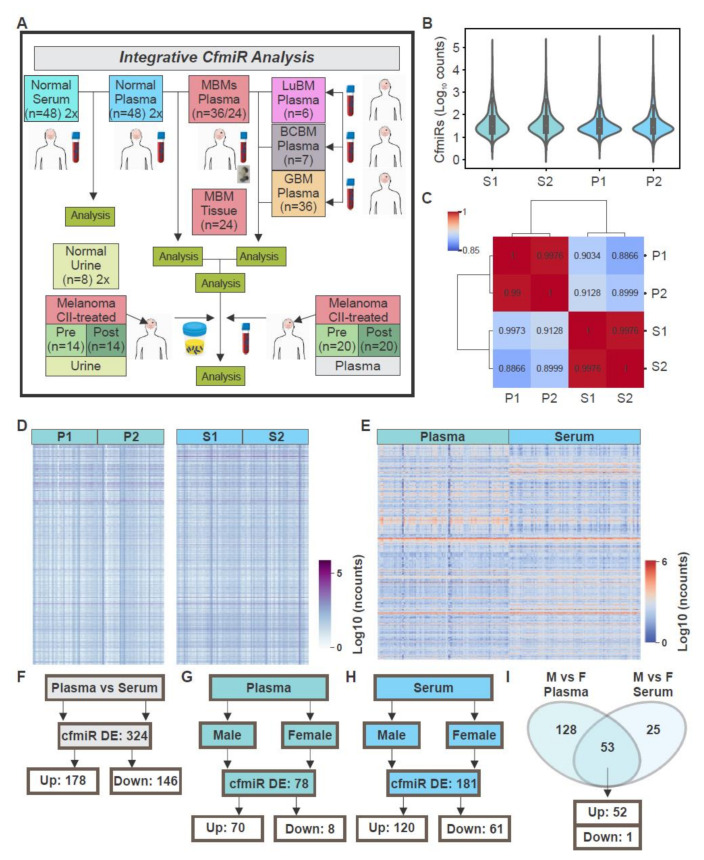
Comparison of serum and plasma samples from normal healthy donors. (**A**) Schematic representation of the samples analyzed throughout the study. (**B**) Cell-free microRNAs (cfmiRs) normalized counts distribution in serum (S1-S2) and plasma (P1-P2) samples for the first (S1 and P1) and second sequencing assays (S2 and P2) respectively. (**C**) Correlation matrix of cfmiRs analyzed in serum (S1-S2) and plasma (P1-P2) samples for the first (S1 and P1) and second sequencing assays (S2 and P2) respectively. (**D**) Heatmap showing the normalized counts of cfmiRs observed in plasma (P1-P2) and serum (S1-S2) respectively. (**E**) Heatmap showing the cfmiRs significantly changing in plasma compared to serum samples. (**F**) Differentially expressed (DE) cfmiRs in plasma versus serum samples. (**G**,**H**) DE cfmiRs in males versus females observed in plasma (**G**) and serum (**H**) samples, respectively. (**I**) Commonly DE cfmiRs in males versus females in serum and plasma samples.

There are various studies on identifying cfmiRs in cancer patients. However, there are limited analyses published for cfmiR profiling of large cohorts of plasma and serum samples taken from normal healthy donors [43]. Initially, we assessed and compared normal donors’ plasma and serum using the HTG miRNA WTA. To determine the reproducibility of the HTG miRNA WTA, normal donors’ plasma (Plasma 1, P1, *n* = 48) and serum (Serum 1, S1, *n* = 48) samples were randomly distributed in a 96-well HTG miRNA WTA. Then, serum (Serum 2, S2, *n* = 48) and plasma (Plasma 2, P2, *n* = 48) samples were obtained from the same healthy donors respectively, and analyzed using HTG miRNA WTA (Figure 1A). Using the parsed raw read counts, we performed DESeq2 data normalization using HTG REVEAL software version 2.0.1 and compared S1 to S2. Normal donors’ serum samples S1 and S2 showed similar counts distributions and a correlation value of 0.99 (Figure 1B,C). Similar comparison and results were observed for plasma samples (P1 and P2, Figure 1B,C). Also, both normal donors’ serum and plasma samples showed high correlation of the cfmiRs expression (Figure 1C). To identify differentially expressed (DE) cfmiRs, normal donors’ plasma and serum samples were compared using HTG REVEAL software version 2.0.1. After data normalization and multiple testing corrections (false discovery rate (FDR) *p* < 0.05), only cfmiRs with a fold-change (FC) of >1.2 or <1.2 and count values of >100 after normalization were included in further analysis. The normal donors’ serum (S1 + S2) and plasma (P1 + P2) samples were pooled and evaluated for differences in cfmiR expression. Overall, the cfmiR profiles were similar for serum and plasma samples, respectively (Figure 1D and Table 1A,B). Only two cfmiRs showed significant changes in the same serum samples analyzed in duplicates (Table 1); while no significant changes were observed for plasma samples analyzed in duplicates. Then, we compared plasma and serum samples. There were 324 DE cfmiRs in plasma compared to serum samples of which 178 were upregulated and 146 were downregulated (Figure 1E,F). Additionally, plasma and serum samples were compared for cfmiRs associated with gender. Plasma samples from males showed 78 DE cfmiRs compared to females, where the majority (89.7% of the total DE cfmiRs) were upregulated in males versus females (Figure 1G). A similar analysis was performed for the comparison in serum samples of both genders. A total of 181 cfmiRs were DE in both genders. While 61 were downregulated, 120 were upregulated in males compared to females (Figure 1H). When considering the top 10 most variable cfmiRs found in plasma, all 10 cfmiRs were upregulated (Table 2A). Similar results were observed when considering the top 10 most DE cfmiRs found in serum, where 9 cfmiRs were upregulated (Table 2B). Finally, we examined for common cfmiRs consistently changing in plasma and serum samples when comparing males versus females. A total of 53 cfmiRs were consistently changing in serum and plasma (Table 3). Of the 53 cfmiRs commonly DE in both comparison, 52 were consistently upregulated in males versus females in both serum and plasma samples (Figure 1I). In summary, the results showed that there were significant differences in plasma and serum cfmiR profiles. There were no significant differences in the cfmiRs profiles analyzed in plasma samples that were run in duplicates demonstrating the robustness and reproducibility of the HTG miRNA WTA. Fewer cfmiRs showed significant differences in gender analysis in plasma compared to serum samples. Subsequent studies on metastatic melanoma patients were performed on plasma because cfmiR detection in serum can be problematic due to clotting event factors that promote the release of miRs from leukocytes and platelets. Also, there is a preferential use of plasma in cfNA clinical assays.

### 2.2. A CfmiR Signature That Identifies MBM Patients from Normal Healthy Donors’ Plasma

**Figure 2:**
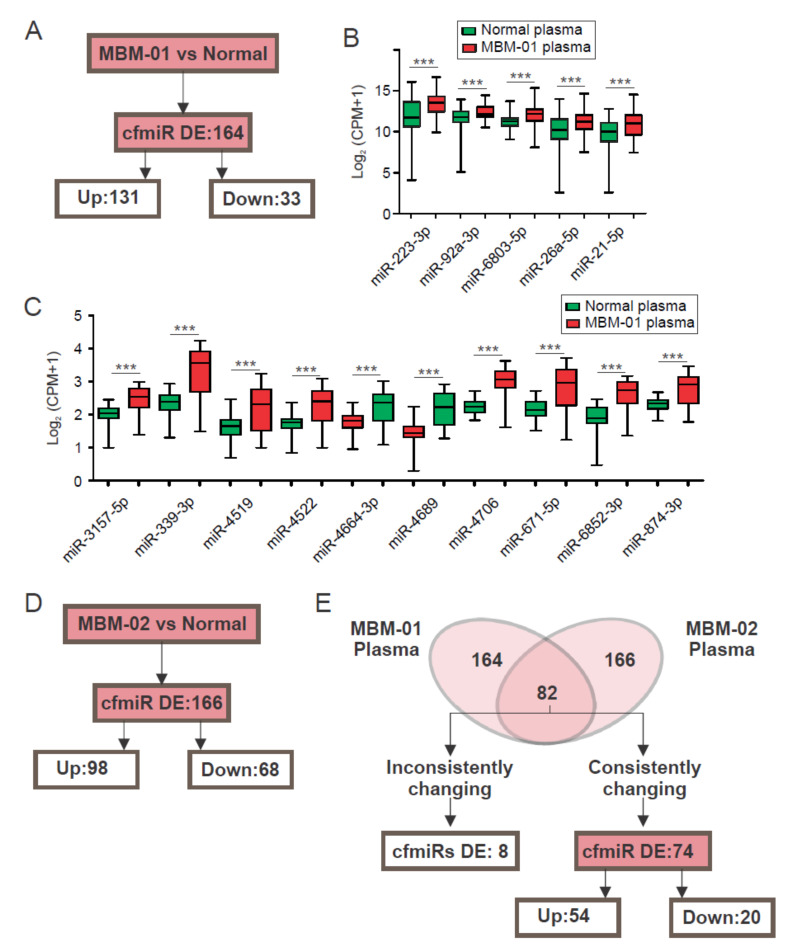
Differentially expressed (DE) cell-free microRNAs (cfmiRs) in serum versus plasma from normal healthy donors’ samples. (**A**) Schematic of the cfmiRs DE, either up- or down-regulated in the plasma of melanoma brain metastasis first cohort (MBM-01) patients compared to the normal donors’ samples. (**B**) Boxplots showing the top 5 DE cfmiRs with the highest detection levels in MBM-01 patients compared to normal donors’ samples (*** *p* < 0.001). (**C**) Boxplots showing the top 10 most DE cfmiR in MBM-01 patients compared to normal donors’ samples (*** *p* < 0.001). (**D**) Schematic of the cfmiRs DE, either up- or down-regulated in the plasma of melanoma brain metastasis second cohort (MBM-02) patients compared to the normal donors’ samples. (**E**) Comparison of the DE cfmiRs and changing inconsistently or consistently in MBM-01 and MBM-02 patients’ cohorts.

MBMs are one of the most devastating and difficult metastases to treat. Thus, earlier MBM detection, identification of progressive disease, and treatment response are critical to improve overall outcomes. All blood cfmiR analysis from this point on will be referring to plasma samples. Pre-operative samples from MBM patients (MBM-01, *n* = 36, Table 4) were assessed to determine cfmiR signatures that could identify the MBM from normal donors’ samples (P1, *n* = 48). For MBM and normal donors’ comparison, the same exclusion criteria was used to exclude cfmiRs with low detectability and that were not significantly changing (FDR *p* < 0.05, FC > 1.2, or <1.2 and count values >100 after normalization). The results showed that 164 cfmiRs were significantly DE in MBM patients’ compared to normal donors’ samples, of which 79.9% (131 of 164) were upregulated, and 20.1% (33 of 164) were downregulated (Figure 2A). In Figure 2B, the top 5 DE cfmiRs with the highest detection level in MBM patients compared to normal donors’ samples are shown. Also, the top 10 most DE cfmiRs are shown (Figure 2C).

To verify the cfmiR signature obtained in the first cohort of MBM patients, we assessed a second cohort of MBM patients (MBM-02, *n* = 24, Table 4). Pre-operative plasma samples from the second cohort of MBM (*n* = 24) patients were assessed and compared to normal donors’ samples (P1, *n* = 48). We observed 166 DE cfmiRs in MBM patients compared to normal donors’ samples (FDR *p* < 0.05, FC > 1.2, or <1.2 and count values >100 after normalization). Of the 166 cfmiRs, 68 were downregulated and 98 were upregulated (Figure 2D). Surprisingly, 74 were consistently changing in both MBM cohorts. While 20 were consistently downregulated, 54 were upregulated (Figure 2E).

**Figure 3:**
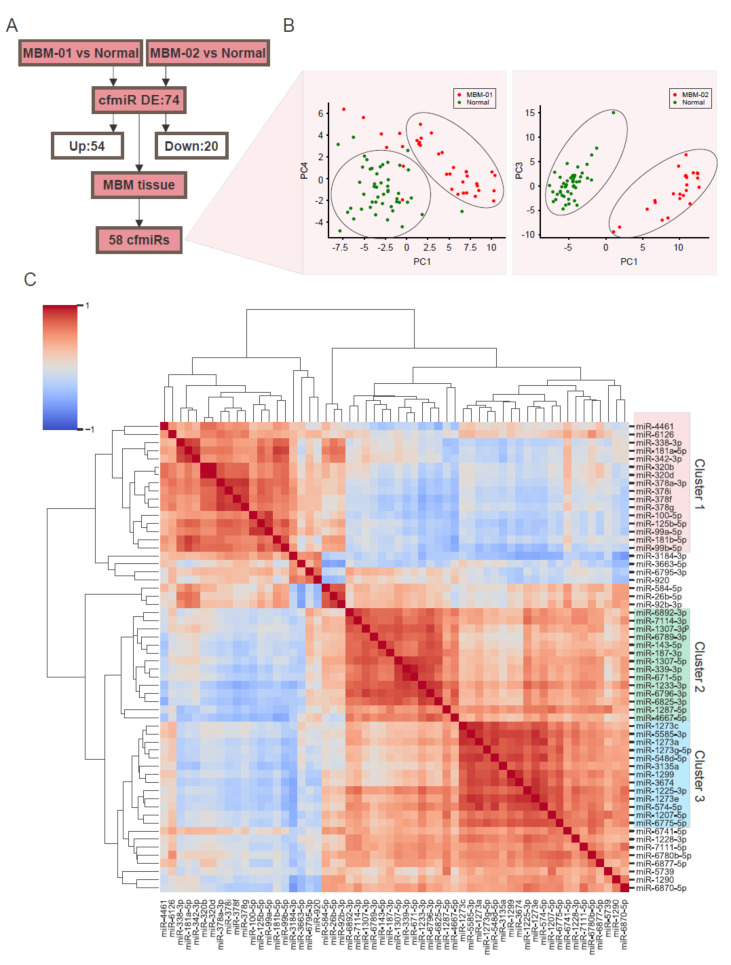
A Cell-free microRNA (cfmiR) signature in the plasma that differentiates melanoma brain metastasis (MBM) patients’ from normal donors’ samples. (**A**) Scheme of the differentially expressed (DE) cfmiRs in MBM patients compared to normal donors’ samples that were detected in MBM tissue samples. (**B**) Principal component analysis (PCA) analysis of the 58 DE cfmiRs in MBM-01 (left) or MBM-02 (right) patients versus normal donors’ samples that were also present in the MBM tissues. (**C**) Clustering of the 58 cfmiRs that differentiate MBM patients from normal donors’ samples. Three clusters were identified: cluster 1 (pink), cluster 2 (green), and cluster 3 (blue).

To corroborate the origin of the MBM cfmiR signature, we analyzed a cohort of MBM tissues (*n* = 24) and compared the 74 cfmiR profiles commonly changing in the two cohorts of MBM patients’ compared to normal donors’ samples. We found 16 (21.6%) cfmiRs changing in MBM plasma that 7 were not present in MBM tissues (Table 5) and 58 (78.4%) DE cfmiRs in MBM plasma that were consistently expressed in the MBM tissues (Figure 3A). This 58 cfmiR signature was verified to differentiate MBM patients from normal donors’ samples using principal component analysis (PCA, Figure 3B). The first five components of the PCA showed 82.5% and 89.1% mean cumulative variance in MBM-01 and MBM-02 respectively (Table 6). To determine the association among the cfmiRs identified, the 58 cfmiRs were clustered based on their correlation values. Three main clusters (cluster 1, 2, and 3) were observed in the correlation matrix (Figure 3C, Table 7A–C). Moreover, two clusters showed that members of the same family clustered together (i.e. cluster 1: miR-320b and miR-320d, miR-181b-5p and miR-181a-5p, miR-99b-5p and miR-99a-5p; cluster 3: miR-1273a, miR-1273c, miR-1273e, and miR-1273g-5p). To summarize, we identified a 74 cfmiR signature differentiating MBM patients’ from normal donors’ samples that were consistently present in the MBM tissues analyzed. These results also validated the hypothesis that there is consistency between the cfmiRs observed in the MBM patients’ plasma and tumors.

### 2.3. CfmiRs That Differentiate MBM from Other Brain Tumors

**Figure 4:**
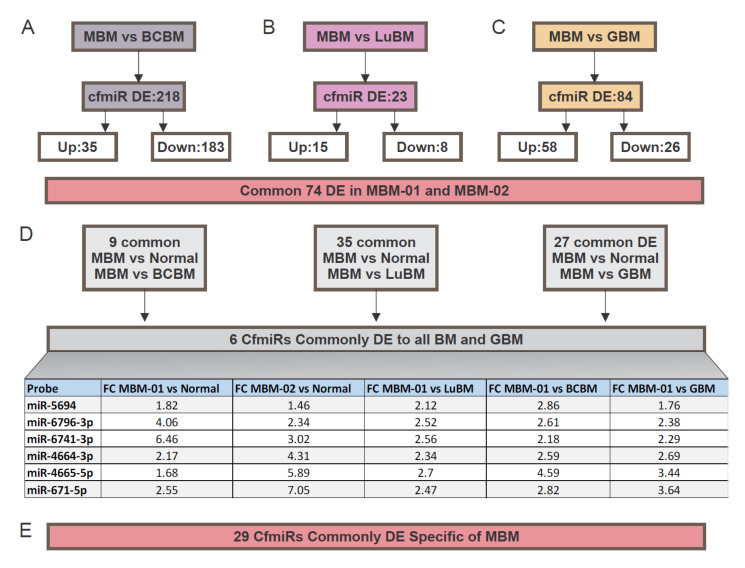
Cell-free microRNAs (cfmiRs) that differentiate melanoma brain metastasis (MBM) patients from extracranial cancer brain metastasis and glioblastoma (GBM) patients. (**A**,**B**) Schematic summary of the cfmiRs identified in MBM vs. breast cancer brain metastasis (BCBM) (**A**), MBM vs. lung cancer brain metastasis (LuBM), (**B**), MBM vs. GBM (**C**). (**D**) A 6 cfmiR signature was identified after comparisons of MBM vs. normal donors to MBM vs. BCBM, MBM vs. LuBM, and MBM vs. GBM plasma samples as summarized in the Table. (**E**) A 29 cfmiR signature was able to distinguish MBM from other primary and metastatic brain tumors. For details on the cfmiRs identified refer to Table 8.

We assessed plasma samples from breast cancer brain metastasis (BCBM) and lung cancer brain metastasis (LuBM), and compared them to MBM patients for identification of distinct cfmiR signatures. BCBM (*n* = 7) and LuBM (*n* = 6) patients samples cfmiR profiles were analyzed and compared to those obtained from the MBM plasma samples (MBM-01, *n* = 36). For this analysis the same exclusion criteria were used (FDR *p* < 0.05, FC > 1.2 or <1.2, and count values >100 after normalization). MBM patients revealed 218 DE cfmiRs compared to BCBM patients (Figure 4A); 35 cfmiR were upregulated and 183 were downregulated in MBM compared to BCBM patients. Similar comparisons were performed in MBM versus LuBM patients. MBM showed that 23 cfmiRs were DE; of these 15 were upregulated and 8 were downregulated (Figure 4B). In summary, 218 and 23 DE cfmiRs were found in the MBM patients when compared to the BCBM and LuBM patients, respectively.

Next, we analyzed for cfmiRs in a cohort of glioblastoma (GBM, *n* = 36) plasma samples. CfmiRs from the first cohort of MBM patients were compared to the cfmiR profiles obtained from the GBM patients. We found that 84 cfmiRs were DE in MBM patients versus GBM patients of which 58 were upregulated and 26 were downregulated (Figure 4C). Through integrated data analysis of the cfmiRs commonly identified as DE between normal donors and MBM (74 DE cfmiRs), BCBM and MBM (218 DE cfmiRs), LuBM and MBM (23 DE cfmiRs), and GBM and MBM (84 DE cfmiRs) plasma samples, only 6 cfmiRs remained commonly DE and detected in all of the comparisons (Figure 4D). These 6 cfmiRs could be used as a potential diagnostic tool to discriminate MBM from LuBM, BCBM, and GBM patients. Then, we searched for cfmiRs that exclusively distinguished MBM patients from normal donors’ samples that were not present in LuBM, BCBM, and GBM patients. The initial 74 cfmiRs, identified in both MBM cohorts, were compared with those DE cfmiRs found in the comparisons of all the respective brain tumor types. An exclusive signature of 29 cfmiRs differentiated MBM from the other brain tumors and normal donors (Figure 4E and Table 8). This 6 cfmiR signature combined with a 29 specific cfmiR signature found in MBM plasma samples may potentially allow for the assessment of melanoma patients developing MBM. More samples are needed to be analyzed in future studies to validate our conclusions.

### 2.4. CfmiR in Patients Treated with Checkpoint Inhibitor Immunotherapy

**Figure 5:**
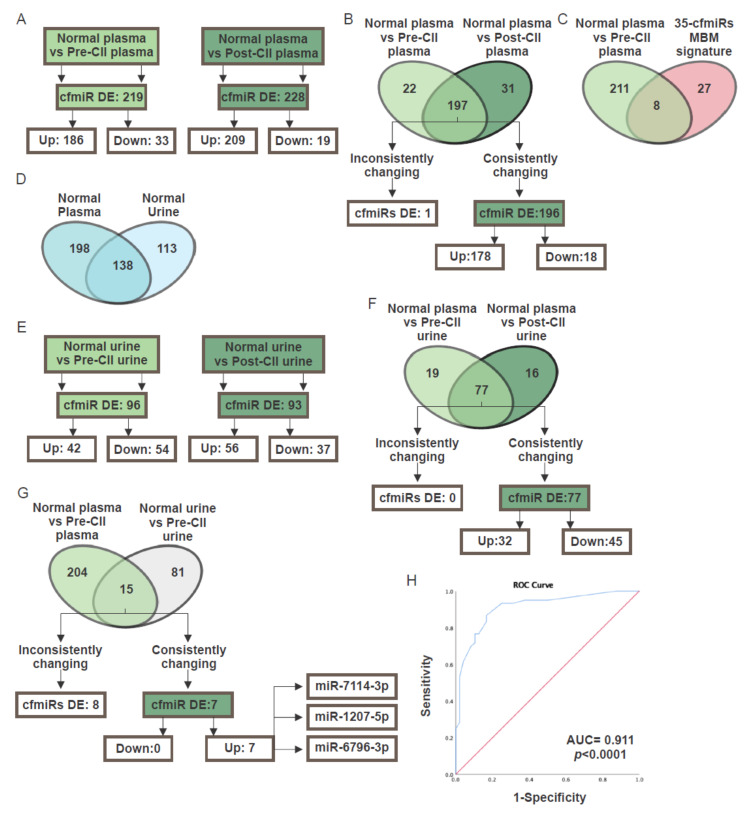
Cell- free miRNA (cfmiR) signatures in urine and plasma of melanoma patients receiving checkpoint immune inhibitor (CII). (**A**) Scheme of the differentially expressed (DE) cell-free microRNAs (cfmiRs) in pre- (left) and post-treatment (right) plasma samples compared to normal donors’ plasma samples. (**B**) CfmiRs detected in plasma samples from pre- and post-treatment melanoma patients receiving CII that were inconsistently or consistently changing. (**C**) Comparison of the 35 cfmiRs melanoma brain metastasis (MBM) signature with the cfmiRs detected in pre-treatment CII plasma samples. (**D**) CfmiRs identified in normal donors’ urine, plasma, or found in both. (**E**) DE cfmiRs detected in urine samples from pre- (left) and post-treatment (right) melanoma patients receiving CII. (**F**) CfmiRs detected in urine samples from pre- and post-treatment melanoma patients receiving CII that were inconsistently or consistently changing. (**G**) DE cfmiRs that were inconsistently and consistently changing in pre-treatment plasma (left) and urine (right) from melanoma patients receiving CII compared to normal plasma and urine respectively. (**H**) Receiver operating characteristic (ROC) curves for the 35 cfmiRs DE in MBM and the associated areas under curves (AUCs).

CII has become the standard of care for metastatic melanoma patients in the last several years and has improved survival outcomes [8]. However, monitoring patients to determine their treatment response remains a major problem. To identify specific cfmiR signatures, in plasma and urine samples from pre- and post-CII-treated melanoma patients (Table 9) were assessed using the HTG miRNA WTA. Plasma samples obtained from both pre- and post-treatment groups were compared to normal donors’ samples. We identified 219 and 228 cfmiRs as DE in pre- and post-treatment CII patients compared to normal donors’ samples, respectively (Figure 5A). A total of 196 cfmiRs were consistently changing in pre- and post-treatment samples. 18 cfmiRs showed a decrease in expression levels, while 178 cfmiRs showed an increase when compared to the normal donors’ samples (Figure 5B). It should be noted that 89.5% of cfmiRs were consistently up or downregulated in the pre- and post-treatment paired samples, respectively. Only 10.5% (22 of 219) of cfmiRs that were detected in pre-treatment samples were not significantly changing in post-treatment samples (data not shown). Also, 8 DE cfmiRs identified in pre- and post-treatment samples were also detected in MBMs but not in other brain cancer metastasis or primary brain tumors (Table 10).

It still remains unknown whether these cfmiRs relate to the clinical course of the disease and if they can be used to monitor a patients’ response to CII-treatment. CfmiRs may be applied to detect immune-related adverse events (IRAE) pre-symptomatically, however, further studies are needed to test this hypothesis.

Consistently, 22.9% (8 of 35) of the DE cfmiRs identified in the two MBM cohorts were also found to be enhanced in the pre-treatment samples of metastatic CII patients (Figure 5C). To summarize, specific cfmiRs are detectable in pre- and post-treatment samples of CII patients. Also, 8 of the 35 cfmiRs MBM signature overlapped with pre-treatment samples of melanoma patients receiving CII.

### 2.5. CfmiR in Urine

CfmiRs in the blood are filtered by the kidney and excreted from the body in the urine. Since the cfmiRs found in the urine are generally diluted, we developed a novel isolation assay to purify and concentrate the cfNA from 15 mL of urine (see Materials and Methods). cfNAs isolated from urine were analyzed using HTG miRNA WTA. Initially, we assessed urine cfmiRs from normal healthy donors (*n* = 8) in duplicates demonstrating reproducibility. The analysis demonstrated that 251 cfmiRs were detected in normal donors’ urine samples using the same cutoff conditions as established for plasma analysis (Figure 5D). 55% (138 of 251) of the cfmiRs detected in normal donors’ urine samples were also present in normal donors’ plasma samples (Figure 5D). Of note, the urine and plasma samples were not isolated from the same patients in these studies. 

On establishment of the urine cfmiR assay, we then analyzed the urine collected from 14 metastatic melanoma patients (Table 9). Normal donors’ urine samples were then compared with pre- and post-treatment urine samples collected from melanoma patients receiving CII. In this analysis 96 cfmiRs were DE in pre-treatment compared to normal donors’ urine samples; while 93 cfmiRs were observed in post-treatment urine samples when compared to normal donors’ urine samples (Figure 5E). In both pre- and post-treatment, 77 cfmiRs were consistently DE (Figure 5F).

In order to determine cfmiRs consistently detected in plasma and urine, 11 metastatic melanoma patients with paired pre-treatment plasma and urine samples were compared. Consistently, 7 cfmiRs were increased in the pre-treatment plasma and urine samples (Figure 5G). Of these 7 cfmiRs, 3 cfmiRs (miR-6796-3p, miR-7114-3p, and miR-1207-5p) were also observed in the 58 cfmiRs detected in MBM plasma and tissues samples and in the 35 cfmiR signature proposed. Surprisingly, miR-7114-3p and miR-1207-5p were also detected in post-treatment plasma (Table 10). Receiver operating characteristic (ROC) curve for the 35 cfmiR signature detected in MBM. Optimal balance between sensitivity (86.7%) and specificity (83.3%) (Area under the ROC curve (AUC) 0.911, 95% CI: 0.855 – 0.966, *p* < 0.001) at 10 cfmiR as a cutoff value (Figure 5H).

## 3. Discussion

Recently cfNA assays have shown promising utility as blood cancer biomarkers particularly when compared to other types of blood cancer biomarkers such as proteins and circulating tumor cells [1,20,23,39,40,44,45,46,47,48]. Many cfNA cancer assays have been Food and Drug Administration (FDA) and Clinical Laboratory Improvement Amendments (CLIA) approved for deciding different cancers’ treatment implementation in the USA and Europe. However, there are still limitations and concerns associated with cfDNA such as the isolation, assay sensitivity, multiple profiling, analytics, and degradation. All of these factors can affect the final results’ interpretation [14,39,40]. To overcome these issues, we utilized a modified HTG miRNA WTA to successfully assess cfmiR found in the plasma and urine samples from cancer patients. 

Our results demonstrated the robustness of the HTG miRNA WTA in detecting cfmiRs in both serum, plasma, and urine samples. The comparison between normal donors’ serum and plasma demonstrated differences in 324 cfmiRs. There are various explanations for this observation, as other groups have shown and discussed previously [14,39,40,44,45]. The limitation in serum analysis is the miRs contamination during blood clotting [39,40]. Differences between serum and plasma could be linked to the presence of extracellular vesicles containing high levels of miRs in the serum [49]. In general, plasma has become the standard blood fluid analytic source for cfNA assays in cancer patient analysis. Our results showed a higher reproducibility in plasma compared to serum, and reduced variability in gender comparisons in plasma. 

A 74 cfmiR signature was detected in two MBM patient plasma cohorts when compared to normal donors’ plasma. Only 58 cfmiRs were consistently detected in MBM tissues. The better explanation for this discrepancy between cfmiRs in MBM patients’ plasma and tumor tissue may arise from differences in the cfmiRs released from the tumor microenvironment, absorption from tumor adjacent normal cells, dilution effect in blood, and filtrations in different organs. Tumor microenvironment contains cells such as immune cells, supporting stroma cells, and/or activated normal brain tissue cells that may account for the release of cfmiRs during brain tumor establishment and progression [36,50].

In the cfmiR comparisons of MBM versus other extracranial origin brain cancer metastasis and GBM, we refined and selected a 6 and 29 cfmiR signature, respectively. The former signature pattern has a potential screening utility to distinguish MBM from other brain metastasis of common tumors and GBM. This signature may have utility in screening patients with brain tumors with an unknown primary tumor. The 29 cfmiR signature in combination with the 6 cfmiR signature may be utilized to monitor MBM patients post-surgery for early disease progression. However, further validation is required for the second signature in an independent cohort of BCBM, LuBM, and GBM patients.

The 35 cfmiR signature identified in MBM were also assessed in pre- and post-treatment plasma samples taken from melanoma patients receiving CII. Interestingly, 8 DE cfmiRs that were exclusively detected in MBM plasma were also detected in the pre- and post-treatment plasma of patients receiving CII. Further validation in a larger independent cohort of melanoma patients receiving CII, with serial blood samples, and Response Evaluation Criteria In Solid Tumors 1.1 (RECIST 1.1) evaluation will be needed in future studies. Successful detection of early progression of CII-treated patients, who have failed treatment, and/or develop MBM will be an important real-time blood diagnostic tool to assist in making treatment decisions for patients.

Urine is an alternative non-invasive body fluid source for assessing cfmiRs. There are several advantages associated with urine-based assays: (1) Non-invasive procedures, (2) can be repetitively monitored, (3) easier compliance by patients, and (4) larger volumes of fluids can be obtained [11]. The procurement of large volumes of blood from CII patients for clinical tests and research purposes is not always feasible. This issue is more prevalent in older patients and in patients who have difficulty having routine blood samples drawn. Conversely, a limitation for cfmiR assessment in urine is the detection of representative cfmiRs derived from tumor cells. Usually cfmiR present in the urine represents those present at high levels in the blood. Many cfmiRs may be filtered out in the kidneys and eventually destroyed in the urine due to pH and other physiological aspects of urine such as metabolic contaminants in urine. Other factors known to influence clinical testing of urine biomarker detection include diurnal fluctuations, time of void collections as related to fluid intake or diuretics, cancer drug treatment, age, etc. Further technical and logistical developments will improve cfmiRs detection in urine. 

Previous studies have identified cfmiRs in different body fluids and have demonstrated how they are distributed [51,52,53]. Ashish et al isolated and sequenced total extracellular RNA from 183 plasma samples, 204 urine samples, and 46 saliva samples from 55 healthy donors, and found that 84 out of 92 miRNAs detected in urine samples were also detected in plasma samples [51]. Our urine analysis showed a similar number of cfmiR detected when compared to plasma. In the present study, we showed that specific cfmiRs identified in the MBM plasma were also detected in the pre- and post-treatment plasma samples from patients receiving CII, and consistently detected in the paired urine samples. 

The urine studies demonstrated the potential utility of the assays to detect cfmiRs in pre- and post-treatment plasma samples from metastatic melanoma patients that may be associated to CII patients’ status. Further studies are needed to increase the sample size and to validate our findings. This study is novel compared to previous studies published in the field of non-urological cancer detection because of: (1) The detection of signatures instead of single biomarkers, (2) the low volumes of blood (<50 µL) required for using a direct assay, (3) the assay relies on an NGS-based platform providing high specificity, (4) the several verification steps that have been included to obtain reproducible cfmiR signatures, and (5) the development of a urine assay enabling cfmiR assessment from melanoma patients. Hence, these findings can be translated into a diagnostic assay for cfmiR monitoring of metastatic melanoma patients. 

## 4. Materials and Methods

### 4.1. Consent to Participate and Patient Specimen Accrual

This pilot study followed the principles in the Declaration of Helsinki. All human samples and clinical information for this study were obtained according to the protocol guidelines approved by the Saint John’s Health Center (SJHC)/John Wayne Cancer Institute (JWCI) Joint Institutional Review Board (IRB): JWCI Universal Consent (Providence Health System Portland IRB: JWCI-18-0401) and Western IRB: MORD-RTPCR-0995. Informed consent was obtained from all participants.

#### 4.1.1. Blood and Urine

Blood samples of healthy donors and cancer patients were obtained at SJHC/JWCI. Blood samples were accrued and processed to obtain serum or plasma. Briefly, all blood samples that were used for plasma isolation were collected in Streck tubes (Streck, La Vista, NE, USA), processed, aliquoted, barcoded, and cryopreserved at −80 °C as previously described [15]. Blood samples that were used for serum isolation were collected in BD Vacutainer® Venous Blood Collection Tubes, BD Diagnostics (VWR, Radnor, PA, USA) and allowed to clot at RT for 1–3 hours, centrifuged, processed as serum, filtered for cell contamination, and cryopreserved at −80 °C as previously described [15]. Aliquots of serum or plasma were thawed only once and mixed, before being analyzed for miR profiling by HTG miRNA WTA. Serum (*n* = 48) and plasma (*n* = 48) were collected from normal healthy donors ranging in age from 21–65 years old and were analyzed twice using HTG miRNA WTA. Single pre-operative blood samples from MBM (*n* = 36, first cohort), MBM (*n* = 24, second cohort), GBM (*n* = 36), BCBM (*n* = 7), and LuBM (*n* = 6) were collected and analyzed using HTG miRNA WTA. Pre- and post-treatment plasma samples from metastatic cutaneous melanoma CII-treated (*n* = 20) patients were also collected and analyzed using HTG miRNA WTA.

Urine samples from healthy donors (*n* = 8) and melanoma patients with paired pre- (*n* = 14) and post-treatment (*n* = 14) urine samples were collected and processed for miR NGS detection as described in Section 4.2.5. Urine samples were collected using a standard sterile 100 mL urine collection cup (Medtronic, Minneapolis, MN, USA). Each urine cup contained Ethylenediamine tetraacetic acid (EDTA) 0.05M pH 8.0 (Bioworld, Little Rock, AR, USA). Total DNA/RNA (~139 ng) was extracted from a 15 mL urine sample using the urine nucleic acid isolation kits and the automated nucleic acid isolation system (JBS Science, Inc., Doylestown, PA, USA). The isolated nucleic acids in elution buffer were then aliquoted and cryostored in a −80 °C freezer until needed for assays.

Eleven patients’ paired urine and blood samples were collected prospectively from melanoma patients. CII treatment responses were assessed at every patient follow-up visit at the cancer clinic as recommended as the standard treatment for the FDA-approved CII drugs (Ipilimumab, Nivolimumab, and Pembrolizumab) [15]. Briefly, computerized tomography/magnetic resonance imaging was assessed every three months according to the Response Evaluation Criteria In Solid Tumors 1.1 (RECIST), denoting progressive disease (PD), stable disease (SD), partial response (PR), complete response (CR), or no evidence of disease (NED). This study was performed in accordance with the Reporting recommendations for tumour marker (REMARK) guidelines [54,55]. The clinical demographics of the melanoma patients analyzed are summarized in Table 4 and Table 9.

#### 4.1.2. MBM FFPE Tissues

MBM tissues were obtained from elective surgeries performed at SJHC. Formalin-fixed paraffin-embedded (FFPE) tissues from MBM patients (*n* = 24) were obtained for HTG miRNA WTA. FFPE MBM tissue sections were cut into 5 µm sections and the tumor areas were micro-dissected with sterile scalpels and dissection needles. Hematoxylin and Eosin (H&E) stained slides marked by a surgical pathologist were used as a guide to determine the area of interest and to avoid including any normal, non-tumor, or necrotic tissue. The tumor tissue section, as determined by H&E, was measured for its total surface area. FFPE tissue sections of approximately 10 mm^2^ were required; multiple slides were micro-dissected for tissue sections less than 10 mm^2^. Sections with tumor tissues exceeding a surface area of 10 mm^2^ were scraped entirely into their respective tubes. Stained H&E slides were overlaid with the unstained slides and then used as templates for micro-dissection. Micro-dissected tissues were processed as described in Section 4.2.3.

### 4.2. Sample Processing for HTG miRNA WTA

For normal donors’ serum/plasma controls, processing and library preparation were performed as described in Section 4.2.1 and Section 4.2.2. For tissue analysis, the samples were processed as described in Section 4.2.3 and Section 4.2.4. Urine processing and library preparation were performed as described in Section 4.2.5.

#### 4.2.1. Serum and Plasma Processing for HTG miRNA WTA

Normal donors’ controls and cancer patients’ serum and plasma samples were thawed, mixed, and analyzed using the HTG miRNA WTA following the manufactures’ recommendations. Serum/plasma (30 µL) were lysed with HTG Biofluids buffer or Plasma Lysis buffer, respectively with Proteinase K (HTG Molecular Diagnostics Inc., Tucson, AZ, USA). Lysed samples were incubated on a heat block at 50 °C for three hours and each hour the samples were mixed by pipetting. Lysed samples were then processed on the automated HTG EdgeSeq instrument for probe-capture of 2083 validated human miRs for 20 hours. After probe-capture, the samples were processed for NGS library preparation.

#### 4.2.2. Serum and Plasma Library Preparation

Probe-captured miR samples were then amplified and indexed via polymerase chain reaction (PCR) using master mix (10 mM deoxyribonucleotide triphosphate (dNTP) 5× Hemo Klentaq® Reaction buffer, Hemo Klentaq® DNA polymerase (New England Biolabs Inc., Ipswich, MA, USA), forward, and reverse primers (HTG Molecular Diagnostics Inc., Tucson, AZ, USA)). The PCR conditions for the reactions were: (1) 95 °C for 4 min, (2) 95 °C for 15 s, (3) 56 °C for 45 s, (4) 68 °C for 45 s, (5) repeat steps 2–4 for 16 total cycles, (6) 68 °C for 10 min, and (7) hold at 4 °C. Following PCR, library cleanup was performed using Ampure^®^ XP beads (Beckman Coulter Inc., Brea, CA, USA). The samples were mixed and incubated with the beads at room temperature (RT) for 5 min. The bead-bound libraries were then washed twice with 80% ethanol to remove any leftover PCR reagents and enzymes. After washing, the samples were dried at RT for 2 min to allow the residual ethanol to evaporate. The libraries were then eluted off the beads with 10 mM Tris hydrochloride (Tris-HCl), pH 8.0. 

#### 4.2.3. MBM Tissue Processing for HTG miRNA WTA

Micro-dissected FFPE tissues were digested in a calculated volume of HTG Lysis buffer equaling 25 µL of Lysis buffer per 10 mm^2^ of total target tissue. HTG denaturation oil was then overlaid on top of each FFPE-Lysis buffer sample. Samples were incubated at 95 °C for 15 min to denature protein structures and remove paraffin wax from the FFPE tissue sections. Samples were cooled down for 10 min at RT and an HTG-provided Proteinase K was pipetted into the aqueous (lysis, non-oil) phase of the samples at a volume 1/20th of the total lysis buffer. The samples were finally incubated at 50 °C for three hours, mixing the aqueous phase by pipetting every hour before loading 25 µL of the sample lysate onto the HTG EdgeSeq instrument for miR probe-capture. 

#### 4.2.4. MBM Tissue Library Preparation

Probe-captured MBM FFPE miR samples were then amplified and indexed via PCR using the master mix (One*Taq* HotStart 2X MasterMix in GC Buffer (New England Biolabs Inc., Ipswich, MA, USA), with forward and reverse primers. PCR conditions were as described in Section 4.2.2. Library cleanup was performed by adding a Clean Up buffer (39% 5M NaCl, 31.25% of 40% PEG 8000, 29.75% molecular-grade water) to AMPure XP beads before combining it with the PCR-amplified sample. Ethanol washing and sample eluting were performed as described above in Section 4.2.2.

#### 4.2.5. MBM Urine Processing for HTG miRNA WTA

After nucleic acid isolation from melanoma patients’ urine samples (refer to Section 4.1.1), 35 ng of RNA was loaded onto the HTG EdgeSeq instrument for WTA probe-capture. Samples were diluted in HTG Lysis buffer or BioFluids Lysis buffer according to the manufacturer’s recommendations. Library preparation and cleanup were as described in Section 4.2.4.

#### 4.2.6. NGS Library Quality Check

All libraries were quantitated using the KAPA Library Quant Kit (Illumina Inc., San Diego, CA, USA) and the Universal qPCR Mix Kit (Roche, Basel, Switzerland) according to the manufacturer’s recommendations. Quality check (QC) for library size was performed on the Agilent Technologies TapeStation 2200 instrument using the High Sensitivity D1000 ScreenTape and High Sensitivity D1000 reagents (Agilent Technologies Inc., Santa Clara, CA, USA). The expected peak size was between 150 and 170 base pairs. Samples that did not indicate proper library formation were excluded from sequencing and repeated for library preparation. 

#### 4.2.7. NGS Library Normalization and Pooling

Quantitated libraries were diluted, normalized, and pooled according to the protocol generated by the HTG EdgeSeq RUO Library calculator software version 2.0.0. Libraries were denatured in 2 N NaOH for 8 min at RT. NaOH was hydrolyzed with 2 N HCl and the denatured pool was diluted down to 20 pM with the HT1 buffer supplied in the MiSeq V3 Kit (Illumina Inc., San Diego, CA, USA). To introduce sequencing diversity and a positive sequencing control, 12.5 pM PhiX Control v3 library (Illumina Inc., San Diego, CA, USA) was spiked into the diluted and denatured 20 pM sample pool. The final pool was made to be 95% sample libraries and 5% PhiX control by concentration. The pooled library was then denatured at 98 °C for 4 min and immediately placed on ice for at least 5 min before loading it onto the Illumina HiSeq 2500, MiSeq, or NextSeq 550 instruments according to the respective Illumina instrument sequencing protocols.

#### 4.2.8. NGS Profiling of the Libraries

Sequencing with Illumina platforms was conducted according to HTG instructions. Sequences were assessed with a read length of 1 × 50 base pairs. FASTQ files were generated from raw sequencing data using Illumina BaseSpace BCL to FASTQ software version 2.2.0 and Illumina Local Run Manager Software version 2.0.0. FASTQ files were analyzed with HTG EdgeSeq Parser software version v5.1.724.4793 to generate raw counts for 2083 miRs per sample.

#### 4.2.9. HTG NGS Arrays

The HTG miRNA WTA was run on a 96-well HTG plate configuration for the normal donors’ plasma and serum samples, while the melanoma patients’ plasma samples were run on a 24-well HTG plate configuration. Each HTG miRNA WTA includes negative (CTRL_ANT1, CTRL_ANT2, CTRL_ANT3, CTRL_ANT4, CTRL_ANT5) and positive (CTRL_miR_POS) miR controls that are included in the 2083 total miR panel. In all runs, Human Brain Total RNA (Ambion, Inc., Austin, TX, USA) was used as a control for library preparation, but they were not sequenced. To test the reproducibility of the HTG miRNA WTA, the normal donors’ plasma and serum patients’ samples were run twice on the HTG EdgeSeq instrument and subsequently sequenced to check for reproducible results.

### 4.3. Biostatistical Analysis

The DESeq2 data subgroup normalization, analyses, and statistical comparisons in: (1) Normal serum versus normal donors’ plasma; (2) MBM-01 plasma versus normal donors’ plasma; (3) MBM-02 versus normal donors’ plasma; (4) MBM01-02 plasma versus MBM tissue; (5) MBM-01 plasma versus LuBM plasma; (6) MBM-01 plasma versus BCBM plasma; (7) MBM-01 plasma versus GBM plasma; and (8) melanoma CII-treated pre- and post-treatment plasma versus normal donors’ plasma; (9) melanoma CII-treated pre- and post-treatment urine versus normal donors’ urine were all performed using the HTG REVEAL software version: 2.0.1. Box plot and t-test analysis were performed with GraphPad prism 5 (GraphPad software Inc., La Jolla, CA, USA). Data processing and analysis were performed using Python 3.7.4 in Jupyter platform [56]. MicroRNA expression counts were logarithmically scaled (log_10_) for data visualization. PCA and data normalization were performed using Scikit-learn open source Python library version 0.22.1 [57] to signify miRNA expression profiles. Matplotlib package version 3.1.3 was utilized for making 2D plots in the python environment [58]. Hierarchically clustered heatmaps were generated using Seaborn version 0.10.1. NumPy library version 1.18.1 [59] and SciPy library 1.4.1 version [60] were utilized to process data matrices. AUC calculation was performed with the SPSS software program, ver. 25.0 (IBM Inc., Chicago, IL, USA). A two-sided *p*-value < 0.05 was considered statistically significant: * *p* < 0.05; ** *p* < 0.01; *** *p* < 0.001 and NS = non-significant. The figures were made using CorelDraw graphics suite 8X (Corel Corporation, Ottawa, ON, Canada).

### 4.4. Data Deposit

The data generated and discussed in this pilot study has been deposited in the National Center for Biotechnology Information’s Gene Expression Omnibus (GEO) and is accessible through the GEO Series accession number GSE150956.

## 5. Conclusions

There is an urgent need to identify robust cfmiR biomarkers that are detectable in body fluid biopsy assays. We have provided a comprehensive analysis (Graphical abstract) using different cohorts of MBM, LuBM, BCBM, and GBM tumors. The results demonstrated how specific cfmiR signatures distinguished MBMs from other types of brain cancer metastases as well as primary GBMs. Moreover, cfmiR detection can potentially be used to monitor melanoma patients undergoing CII therapy. Specific cfmiRs identified in MBMs overlapped with melanoma patients receiving CII. A urine cfmiR assay was developed to demonstrate the detection of cfmiRs and its potential in assessing melanoma patients receiving CII. Our results showed the potential utility of the HTG miRNA WTA and the cfmiR signatures detected in plasma and urine samples for monitoring metastatic melanoma patients’ progression and treatment.

## Figures and Tables

**Table 1:** DE cfmiRs in the plasma (P1 vs. P2) and serum (S1 vs. S2) samples of normal donors.

(A) Top 10 DE cfmiRs expressed in duplicated plasma samples P1 and P2					
Probe	Mean Normalized Expression P1	Mean Normalized Expression P2	FC P1 vs. P2	*p*-Value	Adjusted *p*-Value
miR-6873-3p	99	133	1.21	0.030	0.999
miR-92b-3p	269	369	1.17	0.081	0.999
miR-1273e	350	454	1.16	0.09	0.999
miR-4274	120	109	−1.09	0.138	0.999
miR-1909-5p	144	163	1.11	0.142	0.999
miR-584-5p	103	141	1.14	0.147	0.999
miR-3157-5p	101	115	1.1	0.179	0.999
miR-6852-3p	95	115	1.12	0.185	0.999
miR-572	171	192	1.1	0.187	0.999
miR-1287-5p	371	430	1.11	0.190	0.999
**(B) Top 10 DE cfmiRs expressed in duplicated serum samples S1 and S2**					
**Probe**	**Mean Normalized** **Expression S1**	**Mean Normalized** **Expression S2**	**FC S1 vs. S2**	***p*-Value**	**Adjusted *p*-Value**
miR-6819-5p	149	103	1.36	0.0001	0.027
miR-670-3p	189	123	1.39	0.0001	0.030
miR-1303	181	118	1.37	0.000	0.064
miR-1273e	2776	2058	1.26	0.009	0.418
miR-193a-5p	117	89	1.24	0.012	0.478
miR-1290	229	160	1.26	0.017	0.555
miR-1299	527	381	1.25	0.023	0.562
miR-4769-3p	120	102	1.15	0.027	0.588
miR-6852-5p	106	121	−1.13	0.028	0.588
miR-3674	473	357	1.21	0.048	0.703

DE = differentially expressed. CfmiRs = cell-free microRNAs. FC = fold-change. P1 = plasma 1. P2 = plasma 2. S1 = serum 1. S2 = serum 2. Highlighted in gray are the cfmiRs that were DE in serum samples.

**Table 2:** The top 10 DE cfmiRs in male (M) versus female (F) in normal donors’ plasma (A) and serum (B) samples.

(A) Plasma			(B) Serum		
Probe	FC M vs. F	Adjusted *P*-value	Probe	FC M vs. F	Adjusted *p*-Value
miR-451a	2.29	2.94 × 10^−5^	miR-143-5p	1.34	1.50 × 10^−3^
miR-150-5p	1.84	1.10 × 10^−3^	miR-3943	1.45	2.20 × 10^−3^
miR-6796-3p	1.45	1.90 × 10^−3^	miR-8071	1.45	2.80 × 10^−3^
miR-4726-3p	1.37	2.00 × 10^−3^	miR-4746-5p	1.38	3.30 × 10^−3^
miR-3940-5p	1.59	2.00 × 10^−3^	miR-217	1.38	3.30 × 10^−3^
miR-3155b	1.42	2.10 × 10^−3^	miR-4758-5p	−1.33	4.40 × 10^−3^
miR-6798-5p	1.61	2.10 × 10^−3^	miR-6787-5p	1.38	4.40 × 10^−3^
miR-486-5p	1.70	2.10 × 10^−3^	miR-34b-3p	1.34	5.20 × 10^−3^
miR-3912-5p	1.39	2.50 × 10^−3^	miR-4522	1.42	5.20 × 10^−3^
miR-6789-3p	1.30	2.70 × 10^−3^	miR-193a-3p	1.43	5.20 × 10^−3^

DE = differentially expressed. CfmiRs = cell-free microRNAs. M =male. F = Female. FC = fold-change.

**Table 3:** CfmiRs that are commonly DE in male (M) versus female (F) in both serum and plasma of normal donors’ samples.

Probe	FC Plasma M vs. F	Adjusted *p*-Value	FC Serum M vs. F	Adjusted *p*-Value
miR-451a	2.29	2.94 × 10^−5^	1.41	0.047
miR-150-5p	1.84	1.10 × 10^−3^	1.44	0.023
miR-8071	1.64	3.80 × 10^−3^	1.45	0.003
miR-363-3p	1.58	1.16 × 10^−2^	1.35	0.047
miR-6852-3p	1.56	4.60 × 10^−3^	1.47	0.007
miR-6870-3p	1.52	8.70 × 10^−3^	1.31	0.035
miR-92b-3p	1.49	3.57 × 10^−2^	1.39	0.0387
miR-764	1.48	4.70 × 10^−3^	1.26	0.044
miR-339-3p	1.48	1.75 × 10^−2^	1.37	0.025
miR-6825-3p	1.48	1.05 × 10^−2^	1.43	0.015
miR-6085	1.47	1.17 × 10^−2^	1.38	0.015
miR-6796-3p	1.45	1.90 × 10^−3^	1.24	0.046
miR-3155b	1.42	2.10 × 10^−3^	1.28	0.015
miR-6742-5p	1.42	7.00 × 10^−3^	1.29	0.018
miR-4309	1.42	5.10 × 10^−3^	1.29	0.014
miR-1233-3p	1.42	1.03 × 10^−2^	1.36	0.015
miR-4793-5p	1.42	3.20 × 10^−3^	1.37	0.009
miR-497-3p	1.41	7.00 × 10^−3^	1.32	0.014
miR-6069	1.40	7.00 × 10^−3^	1.3	0.016
miR-640	1.40	8.60 × 10^−3^	1.33	0.012
miR-5694	1.39	9.80 × 10^−3^	1.24	0.039
miR-193b-3p	1.39	1.99 × 10^−2^	1.28	0.011
miR-3912-5p	1.39	2.50 × 10^−3^	1.31	0.011
miR-4655-3p	1.38	4.40 × 10^−3^	1.21	0.039
miR-4726-3p	1.37	2.00 × 10^−3^	1.29	0.010
miR-6715b-3p	1.37	5.10 × 10^−3^	1.31	0.009
miR-3912-3p	1.37	7.50 × 10^−3^	1.32	0.011
miR-3124-3p	1.36	6.10 × 10^−3^	1.26	0.015
miR-33b-5p	1.36	1.66 × 10^−2^	1.29	0.020
miR-671-5p	1.36	1.63 × 10^−2^	1.31	0.039
miR-5587-3p	1.35	1.16 × 10^−2^	1.26	0.027
miR-541-3p	1.35	7.90 × 10^−3^	1.27	0.036
let-7g-3p	1.35	7.00 × 10^−3^	1.3	0.011
miR-4291	1.35	1.75 × 10^−2^	1.32	0.014
miR-2115-5p	1.33	5.40 × 10^−3^	1.23	0.039
miR-1307-5p	1.33	3.90 × 10^−2^	1.33	0.024
miR-3157-5p	1.33	1.15 × 10^−2^	1.36	0.009
miR-217	1.33	1.40 × 10^−2^	1.38	0.003
miR-937-3p	1.32	6.50 × 10^−3^	1.2	0.040
miR-187-5p	1.32	1.16 × 10^−2^	1.3	0.010
miR-4690-5p	1.31	2.70 × 10^−2^	1.25	0.013
miR-187-3p	1.31	1.50 × 10^−2^	1.33	0.01
miR-3907	1.30	2.90 × 10^−3^	1.22	0.03
miR-6789-3p	1.30	2.70 × 10^−3^	1.27	0.01
miR-2115-3p	1.30	7.70 × 10^−3^	1.28	0.011
miR-4279	1.30	2.73 × 10^−2^	1.29	0.014
miR-3714	1.30	1.30 × 10^−2^	1.34	0.005
miR-1909-5p	1.29	1.45 × 10^−2^	1.26	0.022
miR-4537	1.29	7.00 × 10^−3^	1.27	0.011
miR-3689d	1.27	1.65 × 10^−2^	1.31	0.007
miR-935	1.22	3.85 × 10^−2^	1.23	0.015
miR-4746-5p	1.21	4.53 × 10^−2^	1.38	0.003
miR-1273h-5p	−1.51	3.56 × 10^−2^	−1.33	0.034

CfmiRs = cell-free microRNAs. DE = differentially expressed. M = male. F = Female. FC = fold-change.

**Table 4:** Clinical demographics of melanoma brain metastasis (MBM) patients analyzed.

	MBM-01	MBM-02
Variables	*n* (%)	*n* (%)
Age at diagnosis, year, mean (SD)	49 (26)	55 (14)
Age at MBM diagnosis, year, mean (SD)	52 (28)	60 (13)
Gender		
Male	24 (66.7)	14 (58.3)
Female	12 (33.3)	10 (41.7)

MBM-01 = melanoma brain metastasis first cohort. MBM-02 = melanoma brain metastasis second cohort. SD = standard desviation.

**Table 5:** CfmiRs that are commonly DE in MBM-01 and MBM-02 plasma samples compared to normal donors’ samples not present in the MBM tissues.

Probe	FC MBM-01 vs. Normal	Adjusted *p*-Value	FC MBM-02 vs. Normal	Adjusted *p*-Value
miR-6741-3p	6.46	7.65 × 10^−37^	3.02	6.24 × 10^−15^
miR-6852-3p	5.34	2.41 × 10^−22^	4.9	7.63 × 10^−26^
miR-4689	4.14	1.21 × 10^−27^	6.84	4.66 × 10^−41^
miR-3943	3.56	6.57 × 10^−21^	3.01	2.44 × 10^−21^
miR-541-3p	3.34	8.31 × 10^−18^	1.56	7.64 × 10^−4^
miR-3157-5p	3.12	2.07 × 10^−17^	3.27	1.08 × 10^−24^
miR-4784	2.44	1.27 × 10^−13^	1.73	3.33 × 10^−7^
miR-4279	2.36	2.28 × 10^−9^	1.79	5.84 × 10^−6^
miR-4664-3p	2.17	1.96 × 10^−10^	4.31	7.29 × 10^−24^
miR-4291	1.93	1.26 × 10^−5^	2.63	2.68 × 10^−19^
miR-3912-5p	1.88	7.34 × 10^−7^	1.71	1.14 × 10^−7^
miR-5694	1.82	2.47 × 10^−4^	1.46	6.00 × 10^−3^
miR-3912-3p	1.79	1.35 × 10^−5^	1.57	4.39 × 10^−5^
miR-670-3p	1.78	3.53 × 10^−5^	2.66	3.32 × 10^−22^
miR-4665-5p	1.68	2.79 × 10^−4^	5.89	2.77 × 10^−23^
miR-3937	1.67	1.10 × 10^−3^	2.98	3.76 × 10^−8^

DE = differentially expressed. CfmiRs = cell-free microRNAs. MBM-01 = melanoma brain metastasis first cohort. MBM-02 = melanoma brain metastasis second cohort. MBM = Melanoma brain metastasis. FC = fold-change.

**Table 6:** Statistics of PCA analysis of Figure 3B.

	MBM-01 Versus Normal Plasma			MBM-02 Versus Normal Plasma		
PCA Components	Standard Deviation	Proportion of Variance	Cumulative Proportion	Standard Deviation	Proportion of Variance	Cumulative Proportion
PC1	4.906	0.310	0.310	7.011	0.457	0.457
PC2	4.383	0.248	0.558	4.782	0.213	0.670
PC3	3.438	0.152	0.711	3.676	0.126	0.795
PC4	2.452	0.078	0.788	2.458	0.056	0.851
PC5	1.686	0.037	0.825	2.061	0.039	0.891

PCA = principal components analysis. MBM-01 = melanoma brain metastasis first cohort. MBM-02 = melanoma brain metastasis second cohort. PC = principal components.

**Table 7:** DE cfmiRs in MBM-01 and MBM-02 vs. normal donors’ plasma that were also detected in MBM tissues. Cluster 1 (**A**), 2 (**B**), and 3 (**C**) (Figure 3C).

(A) Cluster 1			
Probe	Mean Normalized Expression MBM-01 Plasma	Mean Normalized Expression MBM-02 Plasma	Mean Normalized Expression MBM Tissue
miR-100-5p	150	79	98
miR-125b-5p	235	134	126
miR-181a-5p	1499	842	969
miR-181b-5p	191	113	119
miR-320b	4407	2417	3146
miR-320d	2445	1617	1685
miR-338-3p	224	130	112
miR-342-3p	413	227	285
miR-378a-3p	210	139	126
miR-378f	136	91	82
miR-378g	181	130	118
miR-378i	217	152	133
miR-4461	3221	1837	1113
miR-6126	108,094	78,636	79,367
miR-99a-5p	381	146	195
miR-99b-5p	267	171	121
**(B) Cluster 2**			
**Probe**	**Mean Normalized** **Expression MBM-01 Plasma**	**Mean Normalized** **Expression MBM-02 Plasma**	**Mean Normalized** **Expression MBM Tissue**
miR-1228-3p	92	127	171
miR-1233-3p	234	997	1573
miR-1307-3p	1067	1554	2763
miR-1307-5p	787	2465	1792
miR-143-5p	90	114	239
miR-187-3p	135	185	282
miR-339-3p	289	4295	1437
miR-4667-5p	88	160	237
miR-671-5p	162	1274	539
miR-6789-3p	100	147	178
miR-6796-3p	283	681	1513
miR-6825-3p	173	438	586
miR-6892-3p	409	514	808
**(C) Cluster 3**			
**Probe**	**Mean Normalized** **Expression MBM-01 Plasma**	**Mean Normalized** **Expression MBM-02 Plasma**	**Mean Normalized** **Expression MBM Tissue**
miR-1207-5p	478	759	1354
miR-1225-3p	188	462	609
miR-1273a	109	208	308
miR-1273c	171	243	357
miR-1273e	428	1248	1914
miR-1273g-5p	96	129	176
miR-1299	109	342	318
miR-3135a	81	126	216
miR-3674	88	178	224
miR-548d-5p	144	248	337
miR-5585-3p	1372	2369	3112
miR-574-5p	1632	4042	7203
miR-6775-5p	143	263	279

DE = differentially expressed. CfmiRs = cell-free microRNAs. MBM-01 = melanoma brain metastasis first cohort. MBM-02 = melanoma brain metastasis second cohort. MBM = Melanoma brain metastasis.

**Table 8:** 29 DE cfmiRs in MBM patients’ plasma.

Probe	FC MBM-01 vs. Normal	Adjusted *p*-Value	FC MBM-02 vs. Normal	Adjusted *p*-Value
miR-3937	1.67	1.10 × 10^−3^	2.98	2.09 × 10^−9^
miR-1299	2.25	1.07 × 10^−4^	2.95	2.54 × 10^−10^
miR-1273e	3.36	2.30 × 10^−10^	2.79	1.13 × 10^−12^
miR-670-3p	1.78	3.53 × 10^−5^	2.66	2.96 × 10^−24^
miR-1225-3p	2.55	2.52 × 10^−10^	2.42	3.85 × 10^−16^
miR-574-5p	3.27	2.52 × 10^−9^	2.36	3.05 × 10^−7^
miR-6780b-5p	1.90	1.20 × 10^−3^	2.01	6.58 × 10^−7^
miR-3674	1.96	5.90 × 10^−3^	1.95	3.72 × 10^−4^
miR-7111-5p	1.69	6.50 × 10^−3^	1.89	1.30 × 10^−6^
miR-1273a	2.22	7.15 × 10^−6^	1.85	1.06 × 10^−4^
miR-6877-5p	1.53	2.92 × 10^−2^	1.84	2.85 × 10^−5^
miR-6775-5p	1.57	3.50 × 10^−3^	1.81	2.34 × 10^−6^
miR-4279	2.36	2.28 × 10^−9^	1.79	7.18 × 10^−7^
miR-5585-3p	1.77	5.80 × 10^−3^	1.69	3.48 × 10^−4^
miR-548d-5p	1.81	1.29 × 10^−2^	1.66	7.80 × 10^−3^
miR-7114-3p	1.88	1.70 × 10^−6^	1.65	2.27 × 10^−8^
miR-1207-5p	2.23	1.83 × 10^−7^	1.58	1.71 × 10^−5^
miR-3135a	2.12	1.08 × 10^−5^	1.55	6.26 × 10^−4^
miR-6789-3p	1.44	2.20 × 10^−3^	1.46	1.23 × 10^−5^
miR-1273c	1.67	6.20 × 10^−3^	1.41	1.63 × 10^−2^
miR-1228-3p	1.50	2.80 × 10^−3^	1.38	5.43 × 10^−4^
miR-1273g-5p	1.48	2.29 × 10^−2^	1.35	1.77 × 10^−2^
miR-143-5p	2.12	5.05 × 10^−11^	1.26	4.60 × 10^−3^
miR-6795-3p	−1.31	1.43 × 10^−2^	−1.26	7.06 × 10^−4^
miR-6126	–1.63	2.06 × 10^−2^	−1.36	1.90 × 10^−2^
miR-920	–1.64	1.51 × 10^−8^	−1.42	1.18 × 10^−4^
miR-378f	–2.02	4.64 × 10^−10^	−1.5	1.53 × 10^−5^
miR-3663-5p	–2.33	4.44 × 10^−12^	−1.7	2.23 × 10^−5^
miR-3184-3p	−1430.32	1.02 × 10^−105^	−2.07	2.78 × 10^−11^

DE = differentially expressed. CfmiRs = cell-free microRNAs. MBM-01 = melanoma brain metastasis first cohort. MBM-02 = melanoma brain metastasis second cohort. MBM = Melanoma brain metastasis. FC = fold-change.

**Table 9:** Clinical pathological information for metastatic melanoma patients receiving CII analyzed for cfmiRs in plasma and urine samples.

	Plasma (*n* = 20)	Urine (*n* = 14)	Paired Plasma and Urine (*n* = 11)
Variables	*n* (%)	*n* (%)	*n* (%)
Age, year, mean (SD)	61.8 (21.1)	59.7 (24.9)	55 (13)
Gender			
Male	13 (66.7)	9 (64.3)	6 (54.5)
Female	7 (33.3)	5 (35.4)	5 (45.5)
AJCC 8th stages			
III b/c	3 (15)	4 (28.6)	2 (18.2)
IV b/c/d	17 (85)	10 (71.4)	9 (81.8)
*BRAF* mutations			
Positive	12 (60)	9 (64.3)	7 (63.6)
Negative	8 (40)	5 (35.7)	4 (36.4)
Number of metastasis			
1	17 (85)	13 (92.9)	10 (85)
2–3	3 (15)	1 (7.1)	1 (15)

CII = checkpoint immune inhibitor. Cell-free microRNAs = cfmiRs. AJCC 8th stages at the start of the specific CII. SD = standard deviation.

**Table 10:** CfmiRs that were DE expressed in MBM-01 and MBM-02 cohorts that were also detected in pre- and post-treatment samples of metastatic melanoma patients receiving CII.

Probe	FC MBM-01 vs. Normal	FC MBM-02 vs. Normal	FC MBM vs. LuBM	FC MBM vs. BCBM	FC MBM vs. GBM	FC Pre-CII vs. Normal	FC Post-CII vs. Normal
miR-3184-3p	−2.07	−1430.32	NDE	NDE	NDE	−2669.64	−3258.79
miR-143-5p	1.26	2.12	NDE	NDE	NDE	1.81	2.96
miR-6789-3p	1.46	1.44	NDE	NDE	NDE	1.6	2.94
miR-1207-5p	1.58	2.23	NDE	NDE	NDE	1.54	1.62
miR-7114-3p	1.65	1.88	NDE	NDE	NDE	3.07	5
miR-4279	1.79	2.36	NDE	NDE	NDE	2.87	4.74
miR-1225-3p	2.42	2.55	NDE	NDE	NDE	1.4	2.01
miR-670-3p	2.66	1.78	NDE	NDE	NDE	1.45	1.75

DE = differentially expressed. CfmiRs = cell-free microRNAs. MBM-01 = melanoma brain metastasis first cohort. MBM-02 = melanoma brain metastasis second cohort. CII = checkpoint immune inhibitor. MBM = Melanoma brain metastasis. FC = fold-change. LuBM = lung brain metastasis. BCBM = breast cancer brain metastasis. GBM = glioblastoma. NDE = not detected as DE in the comparison.

## Abbreviations

AJCC	American Joint Committee on Cancer
AUC	Areas Under Curves
BCBM	Breast Cancer Brain Metastasis
cfmiR	Cell-free miR
cfDNA	Cell-free DNA
cfNA	Cell-free Nucleic Acids
CII	Checkpoint Inhibitor Immunotherapy
CLIA	Clinical Laboratory Improvement Amendments
dNTP	Deoxyribonucleotide Triphosphate
EDTA	Ethylenediamine Tetraacetic Acid
IRAE	Immune-Related Adverse Events
DE	Differentially Expressed
FC	Fold-Change
FDA	Food and Drug Adminstration
FDR	False Discovery Rate
FFPE	Formalin-Fixed Paraffin-Embedded
GBM	Glioblastoma
GEO	Gene Expression Omnibus
H&E	Hematoxylin & Eosin
LuBM	Lung Cancer Brain Metastasis
MBM	Melanoma Brain Metastasis
miR	MicroRNA
PCA	Principal Component Analysis
PC	Principal Components
PCR	Polymerase Chain Reaction
QC	Quality Check
NS	Non Significant
RECIST	Response Evaluation Criteria In Solid Tumors 1.1
REMARK	Reporting Recommendations For Tumour Marker
ROC	Receiver Operating Characteristic
RT	Room Temperature
SD	Standard Deviation
sLDH	Serum Lactate Dehydrogenase
WTA	Whole Transcriptome Assay

## References

[1] Matthews N.H., Li W.Q., Qureshi A.A., Weinstock M.A., Cho E., Ward W.H., Farma J.M. (2017). Epidemiology of Melanoma. Cutaneous Melanoma: Etiology and Therapy.

[2] Gershenwald J.E., Balch C.M., Soong S.J., Thompson J.F., Balch C.M., Houghton A.N.A.J.S., Soong S.J., Atkins M.B., Thompson J.F. (2009). Prognostic factors and natural history of melanoma. Cutaneous Melanoma.

[3] Nayak L., Lee E.Q., Wen P.Y. (2012). Epidemiology of brain metastases. Curr. Oncol. Rep..

[4] Izraely S., Sagi-Assif O., Klein A., Meshel T., Tsarfaty G., Pasmanik-Chor M., Nahmias C., Couraud P.O., Ateh E., Bryant J.L. (2012). The metastatic microenvironment: Brain-residing melanoma metastasis and dormant micrometastasis. Int. J. Cancer.

[5] Westphal D., Glitza Oliva I.C., Niessner H. (2017). Molecular insights into melanoma brain metastases. Cancer.

[6] Boire A., Brastianos P.K., Garzia L., Valiente M. (2020). Brain metastasis. Nat. Rev. Cancer.

[7] Tawbi H.A., Forsyth P.A., Algazi A., Hamid O., Hodi F.S., Moschos S.J., Khushalani N.I., Lewis K., Lao C.D., Postow M.A. (2018). Combined Nivolumab and Ipilimumab in Melanoma Metastatic to the Brain. N. Engl. J. Med..

[8] Weiss S.A., Wolchok J.D., Sznol M. (2019). Immunotherapy of Melanoma: Facts and Hopes. Clin. Cancer Res. Off. J. Am. Assoc. Cancer Res..

[9] Weinstein D., Leininger J., Hamby C., Safai B. (2014). Diagnostic and prognostic biomarkers in melanoma. J. Clin. Aesthet. Dermatol..

[10] Van Wilpe S., Koornstra R., Den Brok M., De Groot J.W., Blank C., De Vries J., Gerritsen W., Mehra N. (2020). Lactate dehydrogenase: A marker of diminished antitumor immunity. Oncoimmunology.

[11] Lin S.Y., Linehan J.A., Wilson T.G., Hoon D.S.B. (2017). Emerging Utility of Urinary Cell-free Nucleic Acid Biomarkers for Prostate, Bladder, and Renal Cancers. Eur. Urol. Focus.

[12] Bax C., Lotesoriere B.J., Sironi S., Capelli L. (2019). Review and Comparison of Cancer Biomarker Trends in Urine as a Basis for New Diagnostic Pathways. Cancers.

[13] Diefenbach R.J., Lee J.H., Rizos H. (2019). Monitoring Melanoma Using Circulating Free DNA. Am. J. Clin. Dermatol..

[14] Heitzer E., Haque I.S., Roberts C.E.S., Speicher M.R. (2019). Current and future perspectives of liquid biopsies in genomics-driven oncology. Nat. Rev. Genet..

[15] Lin S.Y., Huang S.K., Huynh K.T., Salomon M.P., Chang S.-C., Marzese D.M., Lanman R.B., Talasaz A., Hoon D.S.B. (2018). Multiplex Gene Profiling of Cell-Free DNA in Patients With Metastatic Melanoma for Monitoring Disease. JCO Precis. Oncol..

[16] Goh J.Y., Feng M., Wang W., Oguz G., Yatim S., Lee P.L., Bao Y., Lim T.H., Wang P., Tam W.L. (2017). Chromosome 1q21.3 amplification is a trackable biomarker and actionable target for breast cancer recurrence. Nat. Med..

[17] Leung F., Kulasingam V., Diamandis E.P., Hoon D.S., Kinzler K., Pantel K., Alix-Panabieres C. (2016). Circulating Tumor DNA as a Cancer Biomarker: Fact or Fiction?. Clin. Chem..

[18] Huynh K., Hoon D.S. (2016). Liquid Biopsies for Assessing Metastatic Melanoma Progression. Crit. Rev. Oncog..

[19] Huang S.K., Hoon D.S. (2016). Liquid biopsy utility for the surveillance of cutaneous malignant melanoma patients. Mol. Oncol..

[20] Fleischhacker M., Schmidt B. (2007). Circulating nucleic acids (CNAs) and cancer—A survey. Biochim. Biophys. Acta.

[21] Schwarzenbach H., Hoon D.S., Pantel K. (2011). Cell-free nucleic acids as biomarkers in cancer patients. Nat. Rev. Cancer.

[22] Fleming N.H., Zhong J., da Silva I.P., Vega-Saenz de Miera E., Brady B., Han S.W., Hanniford D., Wang J., Shapiro R.L., Hernando E. (2015). Serum-based miRNAs in the prediction and detection of recurrence in melanoma patients. Cancer.

[23] Mumford S.L., Towler B.P., Pashler A.L., Gilleard O., Martin Y., Newbury S.F. (2018). Circulating MicroRNA Biomarkers in Melanoma: Tools and Challenges in Personalised Medicine. Biomolecules.

[24] Godoy P.M., Barczak A.J., DeHoff P., Srinivasan S., Etheridge A., Galas D., Das S., Erle D.J., Laurent L.C. (2019). Comparison of Reproducibility, Accuracy, Sensitivity, and Specificity of miRNA Quantification Platforms. Cell Rep..

[25] Heitzer E., Perakis S., Geigl J.B., Speicher M.R. (2017). The potential of liquid biopsies for the early detection of cancer. NPJ Precis. Oncol..

[26] Leidinger P., Keller A., Borries A., Reichrath J., Rass K., Jager S.U., Lenhof H.P., Meese E. (2010). High-throughput miRNA profiling of human melanoma blood samples. BMC Cancer.

[27] Van Laar R., Lincoln M., Van Laar B. (2018). Development and validation of a plasma-based melanoma biomarker suitable for clinical use. Br. J. Cancer.

[28] Margue C., Reinsbach S., Philippidou D., Beaume N., Walters C., Schneider J.G., Nashan D., Behrmann I., Kreis S. (2015). Comparison of a healthy miRNome with melanoma patient miRNomes: Are microRNAs suitable serum biomarkers for cancer?. Oncotarget.

[29] Fogli S., Polini B., Carpi S., Pardini B., Naccarati A., Dubbini N., Lanza M., Breschi M.C., Romanini A., Nieri P. (2017). Identification of plasma microRNAs as new potential biomarkers with high diagnostic power in human cutaneous melanoma. Tumour Biol. J. Int. Soc. Oncodevelopmental Biol. Med..

[30] Philippidou D., Schmitt M., Moser D., Margue C., Nazarov P.V., Muller A., Vallar L., Nashan D., Behrmann I., Kreis S. (2010). Signatures of microRNAs and selected microRNA target genes in human melanoma. Cancer Res..

[31] Greenberg E., Besser M.J., Ben-Ami E., Shapira-Frommer R., Itzhaki O., Zikich D., Levy D., Kubi A., Eyal E., Onn A. (2013). A comparative analysis of total serum miRNA profiles identifies novel signature that is highly indicative of metastatic melanoma: A pilot study. Biomarkers.

[32] Bustos M.A., Ono S., Marzese D.M., Oyama T., Iida Y., Cheung G., Nelson N., Hsu S.C., Yu Q., Hoon D.S.B. (2017). MiR-200a Regulates CDK4/6 Inhibitor Effect by Targeting CDK6 in Metastatic Melanoma. J. Investig. Dermatol..

[33] Iida Y., Ciechanover A., Marzese D.M., Hata K., Bustos M., Ono S., Wang J., Salomon M.P., Tran K., Lam S. (2017). Epigenetic Regulation of KPC1 Ubiquitin Ligase Affects the NF-kappaB Pathway in Melanoma. Clin. Cancer Res. Off. J. Am. Assoc. Cancer Res..

[34] Asaga S., Hoon D.S. (2013). Direct serum assay for microRNA in cancer patients. Methods Mol. Biol..

[35] Ono S., Oyama T., Lam S., Chong K., Foshag L.J., Hoon D.S. (2015). A direct plasma assay of circulating microRNA-210 of hypoxia can identify early systemic metastasis recurrence in melanoma patients. Oncotarget.

[36] Hanniford D., Zhong J., Koetz L., Gaziel-Sovran A., Lackaye D.J., Shang S., Pavlick A., Shapiro R., Berman R., Darvishian F. (2015). A miRNA-Based Signature Detected in Primary Melanoma Tissue Predicts Development of Brain Metastasis. Clin. Cancer Res. Off. J. Am. Assoc. Cancer Res..

[37] Gajos-Michniewicz A., Czyz M. (2019). Role of miRNAs in Melanoma Metastasis. Cancers.

[38] Peltier H.J., Latham G.J. (2008). Normalization of microRNA expression levels in quantitative RT-PCR assays: Identification of suitable reference RNA targets in normal and cancerous human solid tissues. RNA (New York, N.Y.).

[39] Lanidou E., Hoon D., Wittner C., Rafai N., Horvath R. (2017). Circulating Tumor Cells and Circulating Tumor DNA as a real time liquid biopsy approach. Tietz Textbook Clinical Chemistry and Moelcular Diagnostics.

[40] Lanidou E., Hoon D., Wittner C., Park S., Rafai N., Horvath R. (2018). Circulating Tumor Cells and Circulating Tumor DNA. Tietz Textbook of Clinical Chemistry and Molecular Diagnostics.

[41] Nziza N., Jeziorski E., Delpont M., Cren M., Chevassus H., Carbasse A., Mahe P., Abassi H., Joly-Monrigal P., Schordan E. (2019). Synovial-Fluid miRNA Signature for Diagnosis of Juvenile Idiopathic Arthritis. Cells.

[42] Trejo C.L., Babić M., Imler E., Gonzalez M., Bibikov S.I., Shepard P.J., VanSteenhouse H.C., Yeakley J.M., Seligmann B.E. (2019). Extraction-free whole transcriptome gene expression analysis of FFPE sections and histology-directed subareas of tissue. PLoS ONE.

[43] Max K.E.A., Bertram K., Akat K.M., Bogardus K.A., Li J., Morozov P., Ben-Dov I.Z., Li X., Weiss Z.R., Azizian A. (2018). Human plasma and serum extracellular small RNA reference profiles and their clinical utility. Proc. Natl. Acad. Sci. USA.

[44] Huang S., Hoon D. (2016). Liquid Biopsy Utility for the Surveillance of Cutaneous Malignant Melanoma Patients. Mol. Oncol..

[45] Huynh K., Hoon D. (2016). Liquid Biopsies for Assessing Metastatic Melanoma Progression. Crit. Rev. Onco..

[46] Nolte F., Best H., Kelley T., Patel J., Lanidou E., Hoon D., Chiu R., Lo D., Wittner C., Park S., Rafai N., Horvath R. (2018). Molecular Applications. Tietz Fundamentals of Clinical Chemistry.

[47] Ono S., Lam S., Nagahara M., Hoon D. (2015). Circulating MicroRNA Biomarkers as Liquid Biopsy for Cancer Patients: Pros and Cons of Current Assays. J. Clin. Med..

[48] Han X., Wang J., Sun Y. (2017). Circulating Tumor DNA as Biomarkers for Cancer Detection. Genom. Proteom. Bioinform..

[49] Skog J., Würdinger T., van Rijn S., Meijer D.H., Gainche L., Sena-Esteves M., Curry W.T., Carter B.S., Krichevsky A.M., Breakefield X.O. (2008). Glioblastoma microvesicles transport RNA and proteins that promote tumour growth and provide diagnostic biomarkers. Nat. Cell Biol..

[50] Anfossi S., Babayan A., Pantel K., Calin G.A. (2018). Clinical utility of circulating non-coding RNAs—an update. Nat. Rev. Clin. Oncol..

[51] Yeri A., Courtright A., Reiman R., Carlson E., Beecroft T., Janss A., Siniard A., Richholt R., Balak C., Rozowsky J. (2017). Total Extracellular Small RNA Profiles from Plasma, Saliva, and Urine of Healthy Subjects. Sci. Rep..

[52] Weber J.A., Baxter D.H., Zhang S., Huang D.Y., Huang K.H., Lee M.J., Galas D.J., Wang K. (2010). The microRNA spectrum in 12 body fluids. Clin. Chem..

[53] Seashols-Williams S., Lewis C., Calloway C., Peace N., Harrison A., Hayes-Nash C., Fleming S., Wu Q., Zehner Z.E. (2016). High-throughput miRNA sequencing and identification of biomarkers for forensically relevant biological fluids. Electrophoresis.

[54] McShane L.M., Altman D.G., Sauerbrei W., Taube S.E., Gion M., Clark G.M., Statistics Subcommittee of the NCI-EORTC Working Group on Cancer Diagnostics (2005). REporting recommendations for tumour MARKer prognostic studies (REMARK). Br. J. Cancer.

[55] Sauerbrei W., Taube S.E., McShane L.M., Cavenagh M.M., Altman D.G. (2018). Reporting Recommendations for Tumor Marker Prognostic Studies (REMARK): An Abridged Explanation and Elaboration. J. Natl. Cancer Inst..

[56] Perez F., Granger B.E. (2007). IPython: A System for Interactive Scientific Computing. Computing in Science and Engg..

[57] Pedregosa F., Varoquaux G., Gramfort A., Michel V., Thirion B., Grisel O., Blondel M., Prettenhofer P., Weiss R., Dubourg V. (2011). Scikit-learn: Machine Learning in Python. J. Mach. Learn. Res..

[58] Hunter J.D. (2007). Matplotlib: A 2D graphics environment. Comput. Sci. Eng..

[59] Oliphant T.E. (2006). A guide to NumPy (Vol. 1).

[60] Oliphant T.E. (2007). Python for Scientific Computing. Comput. Sci. Eng..

